# Galaxolide-contaminated soil and tolerance strategies in soybean plants using biofertilization and selenium nanoparticle supplementation

**DOI:** 10.3389/fpls.2023.1221780

**Published:** 2023-08-24

**Authors:** Riyadh F. Halawani, Fahed A. Aloufi

**Affiliations:** Department of Environment, Faculty of Environmental Sciences, King Abdulaziz University, Jeddah, Saudi Arabia

**Keywords:** actinobacteria, detoxification, galaxolide, photosynthesis, selenium nanoparticles, soybean

## Abstract

The current study aimed to address the response of soybean (*Glycine max*) plants to biofertilization and selenium supplementation treatments under galaxolide contamination of soil. In this regard, a pot experiment was carried out where the soybean plants were treated with the plant growth-promoting Actinobacteria (*Actinobacterium* sp.) as a biofertilizer (PGPB treatment) and/or selenium nanoparticles (Se treatment; 25 mg L^-1^) under two non-polluted and galaxolide-polluted soils (250 mg galaxolide per kg of soil) to assess the modifications in some plant physiological and biochemical traits. Although higher accumulation of oxidative biomarkers, including hydrogen peroxide (+180%), malondialdehyde (+163%), and protein oxidation (+125%), indicating oxidative stress in galaxolide-contaminated plants, an apparent decline in their contents was observed in response to biofertilization/supplementation treatments in contaminated soil, especially. It was mainly related to the higher detoxification of ROS in PGPB- and Se-treated plants under galaxolide-induced oxidative stress, in which the direct ROS-scavenging enzymes (+44 -179%), enzymatic (+34 - 293%) and non-enzymatic (+35 - 98%) components of the ascorbate-glutathione pathway, and antioxidant molecules (+38 - 370%) were more activated than in control plants. In addition, a higher accumulation of detoxification activity markers, including phytochelatins (+32%) and metallothioneins (+79%), were found in the combined treatments (PGPB+Se) under galaxolide contamination. Moreover, combined treatment with PGPB and Se ameliorated the levels of chlorophyll *a* content (+58%), stomatal conductance (+57%), the maximum efficiency of photosystem II (PSII) (+36%), and photorespiratory metabolism (including +99% in glycolate oxidase and +54% in hydroxypyruvate reductase activity) in leaves under galaxolide contamination, which resulted in higher photosynthesis capacity (+36%) and biomass production (+74%) in galaxolide-stressed plants as compared to control group. In conclusion, the application of beneficial Actinobacteria and selenium nanoparticles as biofertilization/supplementation is expected to be useful for improving plant toleration and adaptation against galaxolide contamination.

## 1 Introduction

The excessive industrial usage of synthetic and polycyclic musk compounds, which are usually consumed as aroma additives in household and cosmetics products, resulted in the accumulation of pollutants in the environment (Zhang et al., 2019). Among these pollutants, galaxolide (hexahydro-hexamethyl cyclopentabenzopyran) is recognized as one of the most prevalent organic pollutants in the environment, especially because of its low degradation rates (Lv et al., 2021). Although current environmental concentrations of these contaminants are relatively low, their continual input to the environment indicates that the concentration of galaxolide in the environment will increase in the future (Vimalkumar et al., 2021). It is extensively used in the fragrance industry and can be found in various consumer products such as perfumes, lotions, and detergents. Consequentially, it is increasingly released into the environment and becomes an emerging contaminant (Parolini et al., 2015; Vimalkumar et al., 2021). Accordingly, galaxolide pollution is becoming one of the foremost crises for the soil environment and agricultural sector, which could expose a possible menace to plant production and thus, can be harmful to crops, animals, and humans, especially because no removal strategies have been adopted yet (Madnay et al., 2022). So far, however, there has been little discussion about the toxicity effects of galaxolide on plants, its effect was found through inhibiting seed germination (Jiang et al., 2021), affecting chlorophyll content and lipid peroxidation (Chen et al., 2014), and changing malondialdehyde (MDA) content (An et al., 2009). This indicates a need to understand various perceptions of plants’ physiological and biochemical effects upon exposure to galaxolide, and the employed strategies to ensure the maintenance of cellular integrity (Wang et al., 2023). Previous studies have reported that oxidative damage and consequent shifts in antioxidant systems and metabolic strategies may reflect the severity of galaxolide pollution and the sensitivity of crops to galaxolide, so it was proposed as an indicator for the crop-soil galaxolide pollutants interaction (Madnay et al., 2022; Wang et al., 2023). Such toxicity effects of galaxolide contamination are even more severe on dicotyledonous plants (e.g. legumes like soybean, tomato, potato, etc.) than monocotyledonous plants, based on the threshold of no-effect concentration for galaxolide in crops (Wang et al., 2015).

It has been reported that there are interaction effects between galaxolide contamination and microorganisms in the soil, in which microbial communities, galaxolide content, and plant growth are affected (Zhang et al., 2019; Lv et al., 2021). Moreover, the plant-microorganisms interaction has been suggested as one of the principal biological processes influencing the uptake of toxic environmental pollutants by plants (Saravanan et al., 2020). In this regard, some bacteria, particularly those bacteria belonging to Proteobacteria, Firmicutes, and Actinobacteria, have been identified with the simultaneous ability to resist environmental pollution and stimulate plant growth (Yaghoubi Khanghahi et al., 2019a; Saravanan et al., 2020; Terzano et al., 2021). As for the Actinobacteria phylum, these Gram-positive bacteria include some species that are remarkably involved in plant root colonization and can be competent in sustaining bacterial growth under unfavorable conditions through forming spores (Alexander, 1977; Bulgarelli et al., 2013). Actinobacteria are also known for their plant growth-promoting potential (Yadav et al., 2018; Ahmed et al., 2021) and the ability to produce antibiotics compounds, such as actinomycin and streptomycin (Hussein et al., 2018).

The deficiency of selenium (Se), an essential trace element for plant growth, is considered one of the foremost crises in human health, which results in rising threats of numerous diseases (e.g. cardiomyopathy, congestive heart failure, infertility, etc.) (Liu et al., 2021). Accordingly, soil applications of Se-containing fertilizers are recommended to keep the Se level above the deficiency threshold limit in soil and consequently improve Se levels in crops, especially those included in the diets of humans and animals (Duborská et al., 2022). Nevertheless, providing Se with conventional chemical fertilizers (e.g., Na_2_SeO_4_, K_2_SeO_4_, and BaSeO_4_) became a traditional concern in environmental safety because of the high availability and mobility of Se (Rosenfeld et al., 2017; Albqmi et al., 2023a). This matter conducted studies in presenting and examining a further complex of Se supplements called Se nanoparticles (SeNPs) with greatly higher efficiency and lower toxicity (Liu et al., 2021). Moreover, the use of Se nanoparticle supplementation has specifically acquired notable attention because of its favorable consequences on crop growth and production under different environmental and pollution stresses, proving its linkage with crop stress tolerance (Steinbrenner et al., 2016; Cieschi et al., 2019; Albqmi et al., 2023b).

Evaluation of soybean (*Glycine max*) growth under a wide range of environmental and soil conditions is vital to increasing soybean cultivated area and production in Saudi Arabia. Although soybean is not a native crop in arid and semi-arid regions, it has received considerable attention due to its adaptability and principal use as a food crop for human nutrition, a source of protein, a medicinal plant, and recently as an industrial crop (Kahil et al., 2018). Despite the aforementioned research, there has been little understanding regarding the toxic impacts of galaxolide contamination on plants’ agronomic, physiological, and biochemical traits, as well as the interaction effects of biofertilization and Se nanoparticles on the galaxolide-contaminated plants. Therefore, the current study aimed to unravel some of the mysteries surrounding the actively employed strategies by soybean (*Glycine max*) plants under galaxolide contamination of soil, especially when they were inoculated with plant growth-promoting Actinobacteria and treated with Se nanoparticles supplementation. We hypothesized that individual or combined application of plant growth-promoting Actinobacteria and Se nanoparticles could greatly improve the plant tolerance to galaxolide contamination by promoting the function of photosynthesis apparatus and antioxidant defense machinery in galaxolide-stressed plants as compared to the control ones.

## 2 Materials and methods

### 2.1 Plant materials and experimental setup

The experiment was planned on the basis of a completely randomized design with two factors and three replications. The first factor contained four levels of biofertilization/supplementation, including (i) no biofertilization/no supplementation (Co), (ii) soil treated with Se nanoparticles (Se) only, (iii) biofertilization with plant growth promoting actinobacteria (PGPB) only, and (iv) a combined treatment with PGPB and Se (PGPB+Se). The second factor was galaxolide pollution at two levels, including non-polluted soil (control) and galaxolide polluted soil (at 250 mg galaxolide per kg of soil). The dose of applied galaxolide was based on prior research, in which different concentrations of galaxolide (0–500 mg kg^-1^ soil) were investigated on legume growth (Madnay et al., 2022).

Se treatment was applied by soaking the soybean seeds in a solution including 25 mg L^−1^ of Se nanoparticles for 10 h with constant shaking (IKA KS 501 shaker, Staufen, Germany) at room temperature, and washing thrice with distilled water (Albqmi et al., 2023b). The quantity of used concentration of Se nanoparticles was based on prior research experimenting with various levels of Se nanoparticles, including 0, 10, 25, 50, and 75 mg L^−1^, on the growth of crops (Albqmi et al., 2023b).

Biofertilizer (PGPB) treatment was a consortium of four strains of actinobacteria with great potential in plant growth-promoting, which were previously isolated from the legume fields in the Jouf region (Sakaka, Saudi Arabia), and identified as the genus Streptomyces (AbdElgawad et al., 2020). The bacterial suspension (10^6^ CFU ml^-1^) was used as PGPB treatment. Accordingly, Actinobacteria strains were grown in nutrient broth medium at 29°C for 24 h and then were concentrated by centrifugation (at 2660 g for 15 min). Finally, the obtained pellet was washed and re-suspended in a sterile potassium chloride solution (0.9%, w/v) (Yaghoubi Khanghahi et al., 2022a). The density of this bacterial suspension was adjusted to 10^6^ CFU ml^−1^, corresponding to an optical density at 600 nm equal to 0.6–0.7, and it was used to inoculate the soil before the cultivation and being added to the pots (50 ml) every three weeks (Yaghoubi Khanghahi et al., 2021). Control pots were also treated with the sterile potassium chloride solution.

Soybean seeds were sterilized in a sodium hypochlorite solution (1% v/v) for 10 min (Tian et al., 2020) and planted in a potting mix (Tref EGO substrates, Moerdijk, The Netherlands) in pots (12 cm diameter x 25 cm depth), which were filled with a mixture of loamy soil and organic compost (1:1, v/v, Tref EGO substrates). Plants were kept in a controlled-environment chamber for six weeks, with a constant regime of 25-30°C, 14/10 h day/night photoperiod, 220 μmol m^−2^ s^−1^ photosynthetically active radiation (Nagatoshi and Fujita, 2019). Plant shoot (leaf and stem) tissues sampled six weeks after planting, were placed immediately in liquid nitrogen to quench the metabolism, and kept at −80°C. A part of them was used to determine the fresh and dry weights of the shoot and the remaining was used for subsequent biochemical analysis.

### 2.2 Determination of photosynthetic-related parameters

To study the possible effect of PGPB and Se treatments on plant biomass production, we assessed some photosynthetic traits. Accordingly, the contents of photosynthetic pigments, including chlorophyll *a* (Chl *a*), chlorophyll *b* (Chl *b*), and carotenoids in fresh leaves were determined by measuring the absorbance of the extracted samples at 665.2, 652.4, and 470 nm, respectively (Lichtenthaler and Buschmann, 2001). The youngest fully expanded leaves were also subjected to measure the stomatal conductance (gs) and photosynthesis rate (P_N_) using the LI-COR portable photosynthesis system (LI-COR 6400/XT, USA) in a non-destructive measurement. The maximum efficiency of photosystem II in dark-adapted leaves (Fv/Fm) was also determined using a pulse amplitude modulated fluorometer (PAM–2500, Walz, Germany), in which Fm and Fv are the maximum fluorescence and the variable fluorescence, respectively. Briefly, leaves were acclimated to dark conditions for 30 min using dark leaf clips (DLC-8). Then, the leaves were exposed to a low-intensity light (*<*0.1 *μ*mol photons m^−2^ s^−1^, red light) and a saturating light pulse (*>*8,000 *μ*mol photons m^−2^ s^−1^, white light) to measure the basal fluorescence (F0) and the Fm levels (Yaghoubi Khanghahi et al., 2019b). Glycolate oxidase (GO; EC 1.1. 3.15) activity was assessed spectrophotometrically in the fresh samples by measuring the oxidation of O-dianisidine into a colored O-dianisidine radical cation (Kaundal et al., 2012). The assessment of hydroxypyruvate reductase (HPR; EC 1.1.1.81) in leaves was done using NADH-HPR–NADH in the presence of hydroxypyruvate (Bapatla et al., 2021).

### 2.3 Assessment of stress biomarkers

Oxidative stress induced by galaxolide contamination was assessed in fresh leaf samples. In detail, samples were homogenized in ethanol (80% *v*/*v*), and the extracted samples were tested using the thiobarbituric acid assay, followed by reading the absorbance at 440, 532, and 600 nm to determine the malondialdehyde (MDA) content (Hodges et al., 1999). Hydrogen peroxide (H_2_O_2_) content in leaves was also quantified in trichloroacetic acid (0.1%) based on the xylenol orange method, which relies on peroxide-catalyzed oxidation of Fe^2+^ (AbdElgawad et al., 2016). The protein oxidation (PO) parameter in leaves was determined according to the spectrophotometric measurement of protein carbonyl content at 360 nm (Maiti et al., 2012).

### 2.4 Determination of antioxidant metabolites and enzymes and amino acids content

To attain a better in-depth knowledge of the biochemical strategies in plants in response to PGPB and Se treatments under galaxolide exposure, antioxidant metabolites and enzymes were assessed in the fresh samples. To this aim, fresh leaf samples were homogenized in 1 ml buffer [50 mM potassium phosphate, pH 7.0, 1% (w/v) polyvinyl pyrrolidone (PVP), 0.25% (v/v) Triton X-100, 1 mM phenylmethylsulfonyl fluoride (PMSF), 1 mM ASC] and centrifuged to get a clear supernatant for measuring the activity of the antioxidant enzymes. Accordingly, superoxide dismutase (SOD; EC 1.15.1.1) was measured based on the inhibition of nitroblue tetrazolium (NBT) reduction at 560 nm (Dhindsa et al., 1982). Peroxidase (POX; EC 1.11.1.7) activity was determined by determining the pyrogallol oxidation (Kumar and Khan, 1982). The breakdown of H_2_O_2_ at 240 nm was considered to measure catalase (CAT; EC 1.11.1.6) activity (Aebi, 1984). The estimation of ascorbate peroxidase (APX; EC 1.11.1.11) and glutathione reductase (GR; EC 1.8.1.7) activities was fully described by Murshed et al. (2008). The reduction in NADPH absorption at 340 nm was recorded to determine glutathione peroxidase (GPX; EC 1.11.1.9) activity (Drotar et al., 1985). Reduced glutathione (GSH), reduced ascorbate (ASC), and phytochelatins levels were assessed by HPLC (Hartley-Whitaker et al., 2001; Albqmi et al., 2023b). Glutathione S-transferase (GST; EC 2.5.1.18) activity was determined using 1-chloro-2,4-dinitro-benzene as the substrate, based on the method of Habig et al. (1974). The activity of pyrroline-5-carboxylate synthase (P5CS; EC 1.2.1.41) was measured by hydroxamic acid and reading the absorbance of extracted samples at 534 nm, as fully described by Zhang et al. (1995).

To determine the total polyphenols and flavonoids, 100 mg of fresh leaf samples were extracted with ethanol (80%). The extract was centrifuged at 4°C for 20 minutes at 1400g. Clear supernatants were transferred to new Eppendorf tubes for total flavonoids and total phenolics analysis. Folin–Ciocalteu and aluminum chloride colorimetric assays were used to measure the contents of polyphenols and flavonoids, as fully described by Zhang et al. (2006) and AbdElgawad et al. (2020) respectively. The total antioxidant capacity (TAC) in the fresh leaf samples was quantified through the ferric-reducing antioxidant power method using Trolox as a reference base (Selim et al., 2022). The tocopherol content of fresh leaf samples was measured using HPLC, in which dimethyl tocol was used as an internal standard (AbdElgawad et al., 2015). The proline content of fresh leaf samples was analyzed using a fluorometric HPLC method, after oxidization to 4-amino-1-butanol in the presence of chloramine-T and NaBH4 and conversion to *o*-phthaldialdehyde in the presence of 2-mercaptoethanol (Wu, 1993). Quantitative determination of glycine and serine amino acids in the fresh leaf samples was carried out using a Waters Acquity UPLC-tqd system (Milford, Worcester County MA, USA) equipped with a BEH amide 2.1 × 50 column (Yaghoubi Khanghahi et al., 2022b).

### 2.5 Measurement of galaxolide content in plant

Galaxolide was extracted from the fresh leaf tissue using a mixed solution of *n*-hexane and dichloromethane (1:3) based on the method of Chen et al. (2010). The extracted samples were analyzed by gas chromatography-mass spectrometry (GC–MS) with a JB-5MS capillary column (30 m length x 0.25 mm ID x 0.25 μm film thickness), as fully described by Zhang et al. (2019). Five ng of the internal standard PCB-195 (2,2′,3,3′,4,4′,5,6-octachlorobiphenyl) was used.

### 2.6 Statistical analysis

All statistical analyses, including a two-way analysis of variance (ANOVA) and Tukey HSD (honestly significant difference) test, as well as graph drawing, were performed using the SigmaPlot software. The data was normally distributed according to the Shapiro–Wilk test (*p* > 0.05). Values were presented as the average of three biological replicates ± standard deviation.

## 3 Results

**Figure 1:**
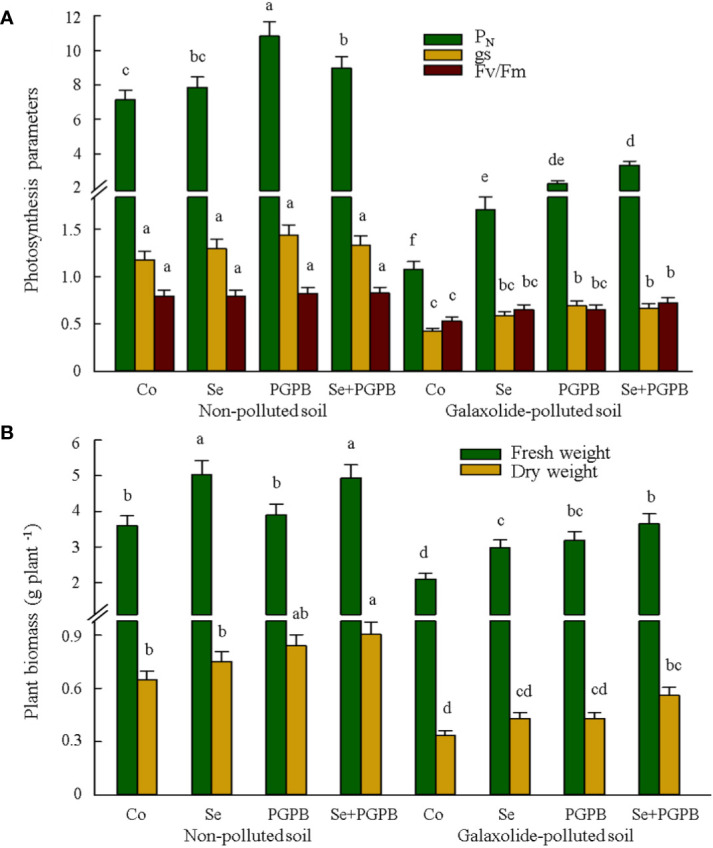
The effect of plant growth-promoting Actinobacteria (PGPB) and Se nanoparticles (Se) on **(A)** the photosynthesis parameters (µmol CO_2_ m^-2^ s^-1^ for P_N_ and mmol CO_2_ m^-2^ s^-1^ for gs) and **(B)** plant biomass in non-polluted and galaxolide-polluted soils. The means in each parameter with a similar small letter(s) are not significantly different at 5% probability level (Tukey test). P_N_: Photosynthesis rate; gs: Stomatal conductance; F_v_/F_m_: maximum efficiency of PSII photochemistry in dark-adapted leaves.

It is apparent from **Figure 1A** that galaxolide-polluted soil caused a sharp decline in the stomatal conductance (gs) and the maximum quantum yield of photosystem II (F_v_/F_m_). Nevertheless, PGPB and Se treatments reduced the intensity of this decline, so that the value of gs and F_v_/F_m_ in the combined treatment (PGPB+Se) under galaxolide contamination was 57% and 36% higher than those contaminated control plants, although it was still 43% and 9% lower than that unfertilized in non-polluted soil (**Figure 1A**). Moreover, PGPB-treated plants had significantly (*p* < 0.05) higher photosynthesis rate (P_N_) in both contaminated and non-contaminated soils equal to 3.3 µmol CO_2_ m^-2^ s^-1^ (PGPB) and 10.8 µmol CO_2_ m^-2^ s^-1^ (PGPB+Se) compared to those in unfertilized plants, which were 1.1 µmol CO_2_ m^-2^ s^-1^ (PGPB) and 7.1 µmol CO_2_ m^-2^ s^-1^, respectively (**Figure 1A**). The results also showed a significant reduction (*p* < 0.05) in plant biomass (dry and fresh weights) in unfertilized plants under galaxolide contamination (**Figure 1B**). Nevertheless, both PGPB and Se treatments significantly (*p* < 0.05) improved the fresh weight (+41-74%) as compared to contaminated control plants (**Figure 1B**). This improvement in dry weight was significant (*p* < 0.05) only in the combined treatment (PGPB+Se) of contaminated plants, which was 68% higher than that in the unfertilized plants (**Figure 1B**).

**Figure 2:**
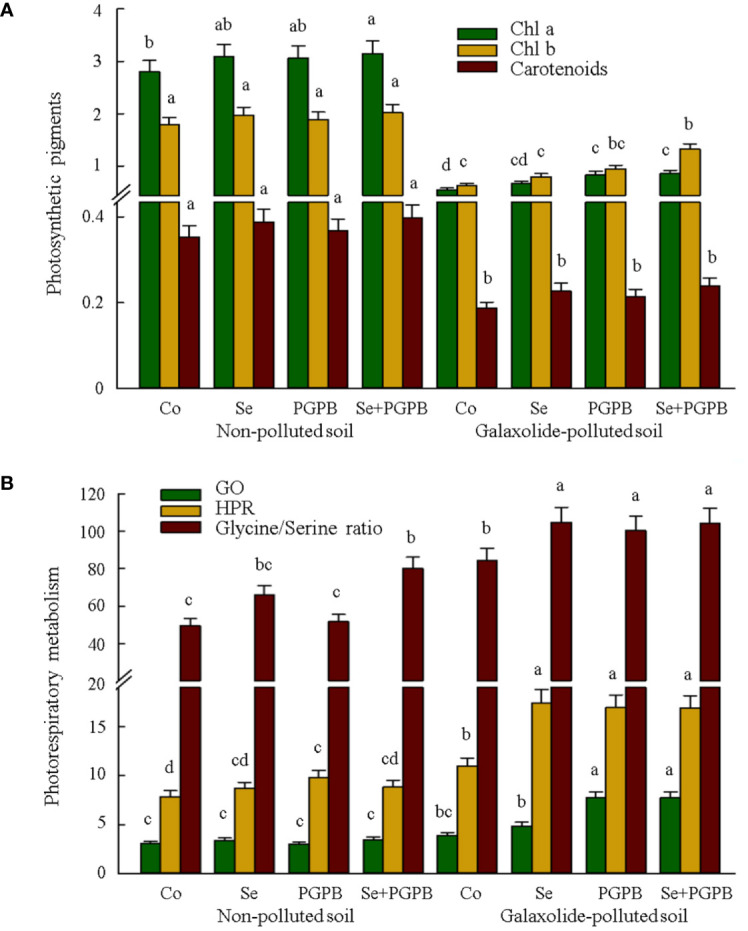
The effect of plant growth-promoting Actinobacteria (PGPB) and Se nanoparticles (Se) on **(A)** the photosynthetic pigments (µg g^-1^) and **(B)** photorespiratory metabolism (μmol mg^-1^ Chl min^-1^ for GO and HPR) in non-polluted and galaxolide-polluted soils. The means in each parameter with a similar small letter(s) are not significantly different at 5% probability level (Tukey test). Chl *a*, Chlorophyll *a*; Chl *b*, Chlorophyll *b*; GO, Glycolate oxidase; HPR, Hydroxypyruvate reductase.

The results of photosynthetic pigments are presented in **Figure 2A**. Under pollution conditions, the concentration of Chl *a*, Chl *b*, and carotenoids was significantly decreased (*p* < 0.05). Nevertheless, the Chl *a* concentration significantly (*p* < 0.05) increased in polluted conditions in PGPB and in PGPB+Se plants; while Chl *b* concentration significantly (*p* < 0.05) increased in polluted conditions just in PGPB+Se treated plants, if compared to the galaxolide-treated control plants (**Figure 2A**). Furthermore, focusing on the photorespiratory metabolism disclosed an obvious increment in the content of glycolate oxidase (+25-99%) and hydroxypyruvate reductase (+54-59%), as well as glycine to serine ratio (+19-24%) in plants treated with PGPB, Se, and PGPB+Se treatments under contamination condition compared to those in unfertilized contaminated plants (**Figure 2B**).

**Figure 3:**
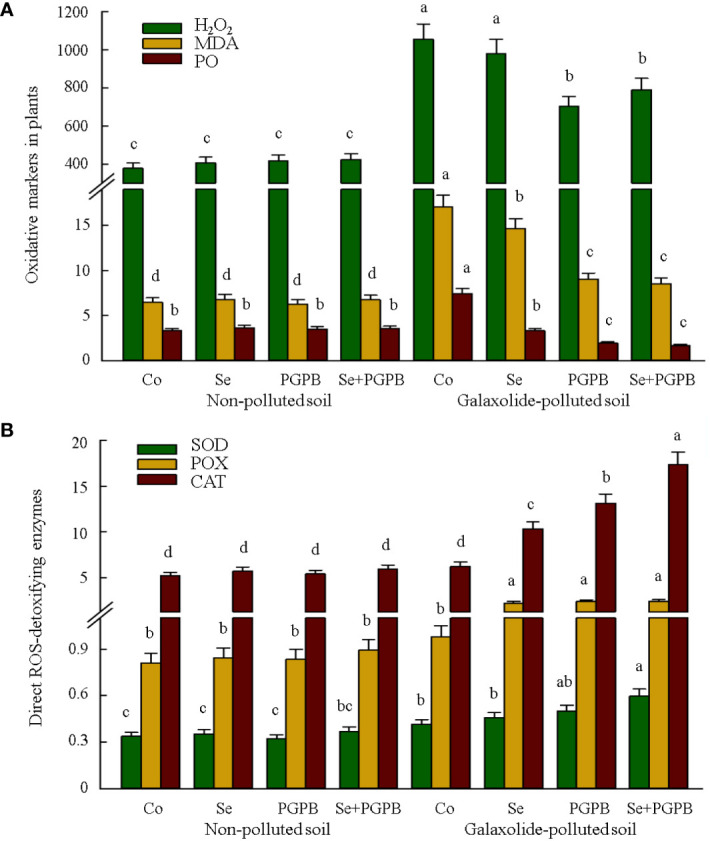
The effect of plant growth-promoting Actinobacteria (PGPB) and Se nanoparticles (Se) on **(A)** the oxidative markers (represented as µmol g^-1^ FW for H_2_O_2_, nmol g^-1^ FW for MDA, and nmol mg^-1^ protein for PO) and **(B)** antioxidant direct-scavenging enzymes (represented as μmol min^-1^ mg^-1^ protein for POX and CAT, and mmol min^-1^ mg^-1^ protein for SOD) in non-polluted and galaxolide-polluted soils. The means in each parameter with a similar small letter(s) are not significantly different at 5% probability level (Tukey test). H_2_O_2_, Hydrogen peroxide; MDA, Malondialdehyde; PO, Protein oxidation; POX, Peroxidase; CAT, Catalase; SOD, Superoxide dismutases.

As shown in **Figure 3A**, strong evidence of oxidative stress was clearly found in plants grown in galaxolide-polluted soil (*p* < 0.05), where the concentrations of oxidative biomarkers, H_2_O_2_, MDA, and PO in no-treated plants in the polluted soil were 2-3 times higher than those in unfertilized control plants in non-polluted soil. Nevertheless, these markers showed a decreasing trend in response to PGPB and Se treatments in stressed plants, especially those PGPB-containing treatments (PGPB and PGPB+ Se). In this regard, the lowest concentration of H_2_O_2_ was recorded in PGPB treatment, while the lowest MDA and PO were in PGPB+Se in the stressed plants, which were about 67%, 50%, and 23% of their concentration in stressed unfertilized plants (*p* < 0.05), respectively (**Figure 3A**).

**Figure 4:**
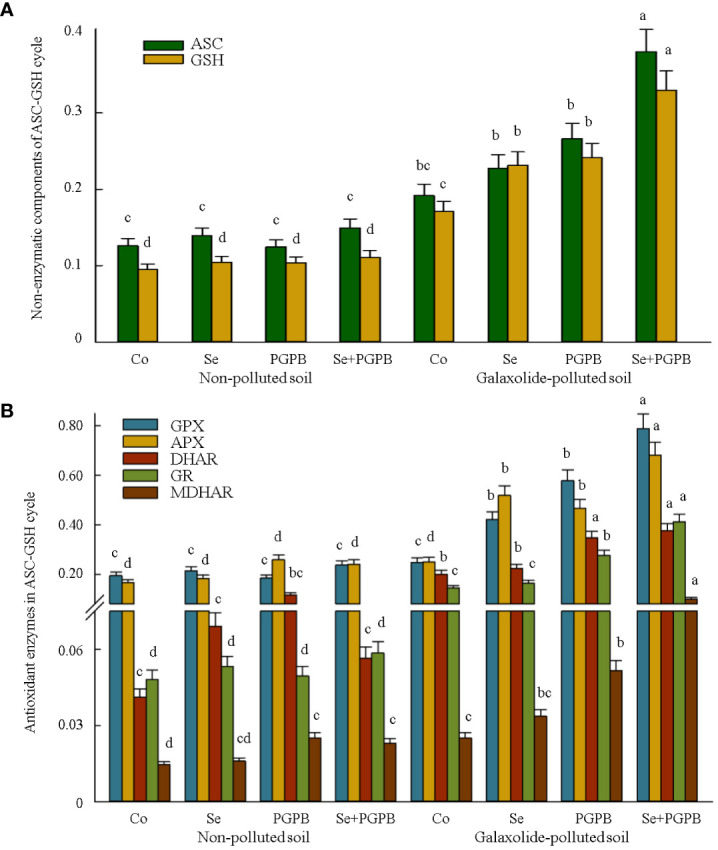
The effect of plant growth-promoting Actinobacteria (PGPB) and Se nanoparticles (Se) on **(A)** the non-enzymatic (µmol g^-1^ FW) and **(B)** enzymatic components (μmol min^-1^ mg^-1^ protein) of the ascorbate-glutathione (ASC/GSH) cycle in non-polluted and galaxolide-polluted soils. The means in each parameter with a similar small letter(s) are not significantly different at 5% probability level (Tukey test). ASC, Ascorbate; GSH, Gluthatione; GPX, Glutathione peroxidase; APX, Ascorbate peroxidase; GR, Glutathione reductase, DHAR, Dehydroascorbate reductase; MDHAR, Mono-dehydroascorbate reductase.

The responses of direct ROS-detoxifying enzymes (CAT, POX, and SOD) and those enzymatic (APX, GPX, GR, DHAR, and MDHAR) and non-enzymatic (ASC and GSH) components of the ascorbate-glutathione (ASC-GSH) pathway were monitored (**Figures 3B**, **4A, B**). Accordingly, among the direct ROS-detoxifying enzymes, only the activity of SOD in unfertilized plants in polluted soil was significantly higher than that in non-polluted soil (*p* < 0.05). Although none of these enzymes were affected by different fertilization treatments under non-polluted conditions, they were greatly activated in plants treated with PGPB, Se, and PGPB+Se treatments in the polluted soil compared to unfertilized plants in non-polluted soils. The greatest activity of SOD, POX, and CAT enzymes was recorded in PGPB+Se treatment, which was significantly (*p* < 0.05) greater than unfertilized plants in the polluted soil, accounting for 43%, 145%, and 179%, respectively (**Figure 3B**).

Similarly, the concentration of non-enzymatic metabolites and enzymatic components of the ASC-GSH cycle was influenced by biofertilization/supplementation treatments in polluted soil (**Figure 4**). The accumulation of ascorbate and glutathione reached the maximum value in the combined treatment (PGPB+Se) in the plants grown in the polluted soil, which was almost two times more than the content in the unfertilized stressed plants (**Figure 4A**). Such improvements in ASC and GSH in the stressed plants clearly resulted in greater increased shifts in the antioxidant enzyme levels in the ASC-GSH cycle. Similarly, the synergistic effect of PGPB and Se treatments had the greatest impact on the activity of GPX, APX, DHAR, GR, and MDHAR enzymes in stressed plants, which was even significantly (*p* < 0.05) greater than the effect of the individual application of these treatments. Accordingly, the activities of GPX, APX, DHAR, GR, and MDHAR in PGPB+Se treatment in contaminated plants were 3.2, 2.8, 1.9, 2.9, and 4 times higher than those in unfertilized stressed plants, respectively (**Figure 4B**).

**Figure 5:**
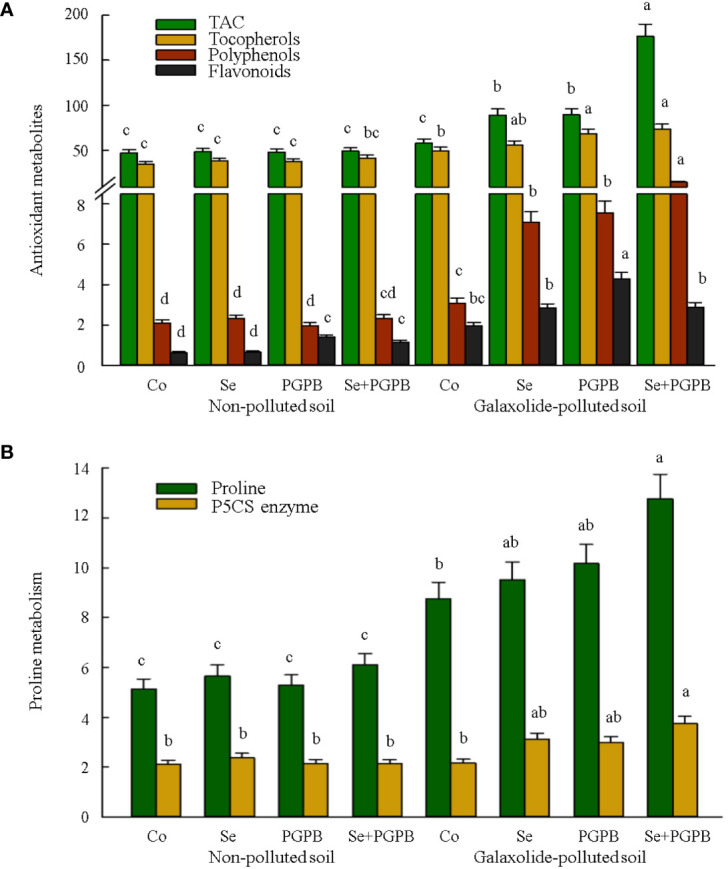
The effect of plant growth-promoting Actinobacteria (PGPB) and Se nanoparticles (Se) on **(A)** the antioxidant metabolites in plant (µmol Torolex g^-1^ FW for TAC; mg GAE g^-1^ FW for polyphenols; mg Quercetin g^-1^ FW for flavonoids; ng g^-1^ for tocopherols) and **(B)** proline metabolism (represented as mg g^-1^ of protein for proline and nmol min^-1^ mg^-1^ protein for P5CS) in non-polluted and galaxolide-polluted soils. The means in each parameter with a similar small letter(s) are not significantly different at 5% probability level (Tukey test). TAC, Total antioxidant capacity; P5CS, Pyrroline-5-carboxylate synthase.

Then the responses of some plant antioxidant metabolites, including tocopherols, polyphenols, flavonoids, and TAC to the galaxolide pollution were investigated (**Figure 5A**). Almost similar responses were also observed for TAC, total tocopherols, and polyphenols, in which the highest contents were recorded in the combined treatment (PGPB+Se) in contaminated plants (**Figure 5A**). Nevertheless, PGPB treatment significantly improved flavonoids in both non-polluted and galaxolide-polluted soils as compared to unfertilized plants (*p* < 0.05). Accordingly, the highest concentration of flavonoids was obtained from PGPB-treated plants in contaminated soil, which was about 2 and 7 times higher than unfertilized plants in contaminated and non-contaminated soils, respectively (**Figure 5A**). Moreover, the proline content was also measured as one of the most critical stress defense molecules in plants. As a result, proline content was significantly (*p* < 0.05) raised in plants when exposed to galaxolide pollution (**Figure 5B**). Such an increment was more pronounced in plants treated with PGPB+Se, equal to 46% and 149% greater than unfertilized plants grown in the polluted and non-polluted conditions (**Figure 5B**). Likewise, PGPB+Se treatment had a maximum activity of pyrroline-5-carboxylate synthase (P5CS) enzyme in contaminated plants were 74% and 76% more activated than unfertilized plants in the polluted and non-polluted soils, respectively (**Figure 5B**).

**Figure 6:**
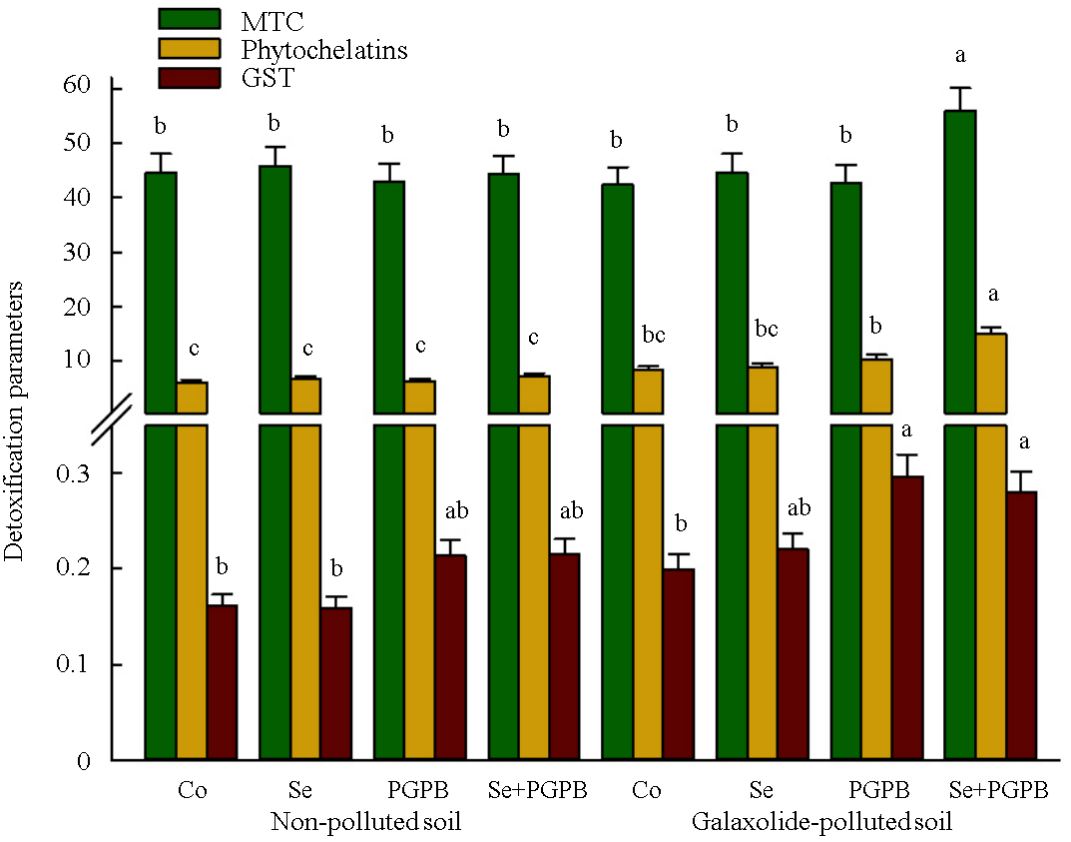
The effect of plant growth-promoting Actinobacteria (PGPB) and Se nanoparticles (Se) on detoxification activity (µg g^-1^ FW) in non-polluted and galaxolide-polluted soils. The means in each parameter with similar small letter(s) are not significantly different at 5% probability level (Tukey test). MTC, Metallothioneins; GST, Glutathione-S-transferase.

Although the individual application of PGPB and Se treatments did not have a significant effect on phytochelatins and metallothioneins (MTC), their simultaneous application led to a significant (*p* < 0.05) improvement of these detoxification parameters (**Figure 6**). In this regard, an increment of 32% and 80% was recorded in the content of phytochelatins and MTC in PGPB+Se treatment in the contaminated soil, respectively, as compared to unfertilized plants grown in the polluted conditions (**Figure 6**). Also, PGPB-treated plants showed the highest content of glutathione-S-transferase (GST) under pollution condition, which were 40% and 73% higher than unfertilized plants under polluted and non-polluted conditions, respectively (**Figure 6**).

**Figure 7:**
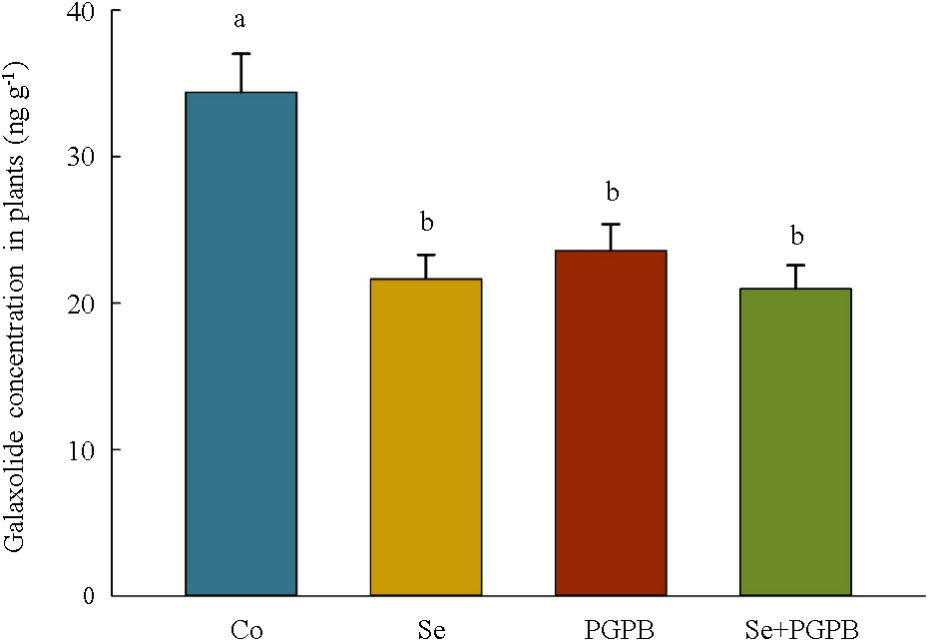
The effect of plant growth-promoting Actinobacteria (PGPB) and Se nanoparticles (Se) on galaxolide concentration in plants grown in galaxolide-polluted soils. The means in each parameter with similar small letter(s) are not significantly different at 5% probability level (Tukey test).

Moreover, the content of galaxolide in plants was significantly (*p* < 0.05) lower in plants treated with PGPB (–31%), Se (–33%), and PGPB+Se (–39%) as compared to unfertilized plants grown in galaxolide-polluted soil (**Figure 7**).

## 4 Discussion

One of the objectives of the present study was to investigate the adverse impacts of galaxolide contamination in soil on soybean and the reaction of the plants to this contamination, especially in response to PGPB and Se nanoparticles supplementation. The decline in the content of photosynthetic pigments and the efficiency of the photosynthesis pathway under environmental pollution-induced stress were already noted by other researchers, who linked this matter with the disturbance of the photosynthetic electron transport chain and the production of reactive oxygen species (ROS) within chloroplasts, subsequently influencing photosynthesis pigment synthesis (Li et al., 2020; Albqmi et al., 2023b). Nevertheless, since the advancement of enzymatic and non-enzymatic antioxidants, metabolites, and detoxification activity under galaxolide contamination, is associated with an improvement in photosynthetic parameters in plants (Zhang et al., 2019; Madnay et al., 2022), it can be suggested that the higher tolerance of plants treated with biofertilization/supplementation, especially those treated with PGPB, to galaxolide pollution conditions led to the improvement of the biosynthesis of photosynthetic pigments and finally a higher rate of photosynthesis compared to the unfertilized plants under pollution conditions. It can be also assumed that the photorespiratory-related parameters were even more pronounced than pigment content in lowering the extent of damages in the photosynthesis rate in fertilized plants compared to those unfertilized under pollution. These findings support the reports of other researchers, who documented advancement in the photosynthesis capacity, stomatal conductance, and pigments concentration in plants treated with Se nanoparticles and beneficial microorganisms under environmental stress and pollution (Habeeb et al., 2020; Madnay et al., 2022; Albqmi et al., 2023b). The decrease in Fv/Fm ratio in galaxolide-stressed plants was also a sign of affecting the function of PSII. This ratio was defined as the highest efficiency of PSII in dark-adapted leaves, usually varying from 0.79 to 0.85 in unstressed plants (Yaghoubi Khanghahi et al., 2019b), which in the present study was in the range of 0.79 to 0.82 in unstressed plants. Nonetheless, the more elevated Fv/Fm ratio in plants treated with combined treatment under galaxolide contamination as compared to unfertilized plants could demonstrate the potential of beneficial Actinobacteria and Se nanoparticles in regulating the portion of excitation energy getting the reaction centers in PSII and avoiding photo-damage in leaves under stress (Yaghoubi Khanghahi et al., 2020). Likewise, previous research indicated that PGPB can systematically induce tolerance in plants against environmental stress and pollution (Meena et al., 2019) through some mechanisms such as the improvement of soil–plant system capability in providing/uptaking essential nutrients from the soil (Yaghoubi Khanghahi et al., 2018), synthesis of indole acetic acid (Strafella et al., 2021), and regulation in overexpression of some specific stress-related genes (Yaghoubi Khanghahi et al., 2022a). Furthermore, dry and fresh weights were significantly decreased in galaxolide-stressed plants. This finding was expected since the photosynthetic machinery and antioxidant defense system were negatively affected by galaxolide contamination in unfertilized plants, as previously reported by Zhang et al. (2019) and Madnay et al. (2022). Therefore, the improvement in the aforementioned parameters resulted in a higher biomass accumulation in response to PGPB and Se.

In supporting these results, the photorespiration pathway, as one of the main sources of ROS in peroxisomes of stressed plants (Bapatla et al., 2021), was also investigated by evaluating glycolate oxidase (GO) and hydroxypyruvate reductase (HPR) activity, as well as gly/ser ratio under galaxolide contamination. In this regard, a significant boost in GO and HPR activity, and gly/ser ratio was found in all biofertilization/supplementation treatments. Such advancement in the content of these key enzymes involved in photorespiratory was in concurrence with previous research under environmental pollution (D’Alessandro et al., 2013), suggesting that photorespiration could donate to the protection of photosynthetic components against over-reduction in oxidative stress conditions through excess energy dissipating process in PSII (Hu et al., 2020; Bapatla et al., 2021). In addition, the greater gly/ser ratio in plants treated with PGPB, Se, and PGPB+Se indicates the significant effects of these treatments in handling nitrogen metabolism in leaves and promoting the fixation of nitrogen into amino acids (Hu et al., 2020), which is therefore vital in managing allocation of excitation energy under oxidative stress conditions (Hu et al., 2014).

Higher accumulation of oxidative biomarkers, including hydrogen peroxide (H_2_O_2_), malondialdehyde (MDA), and protein oxidation (PO), exposed oxidative stress in plants and was caused by galaxolide contamination. This result agrees with the findings of other studies, in which oxidative stress was reported in crops due to galaxolide contamination, mainly because the level of such oxidative stress may exceed the capacity of the antioxidant enzymatic pool (Zhang et al., 2019; Madnay et al., 2022). Moreover, the accumulation of these oxidative markers showed an apparent decline tendency in response to biofertilization/supplementation treatments in contaminated soil, especially in PGPB-treated plants. The findings observed in this study somehow mirror those of the previous studies that have documented the positive effects of PGPB and Se in declining the concentration of oxidative markers in plants when exposed to the contaminations like galaxolide and heavy metals (Zhang et al., 2019; Madnay et al., 2022; Albqmi et al., 2023a). A possible explanation for this result could be a higher detoxification of reactive oxygen species (ROS) in PGPB- and Se-treated plants under galaxolide-induced oxidative stress, in which the antioxidant enzymes and metabolites were more activated than plants grown in the polluted soils. The relationship between detoxifying overproduced ROS in plants and enhancing the antioxidant defense mechanisms was earlier documented (Shabbaj et al., 2022), especially when the plants were inoculated with beneficial bacteria (Madnay et al., 2022). In this regard, it has previously been reported that the crops with higher levels of direct ROS-detoxifying enzymes (POX, SOD, and CAT) and antioxidant enzymes and metabolites involved in the ASC/GSH pathway (ASC, GSH, APX, DHAR, MDHAR, GR, and GPX) can withstand the negative consequences of galaxolide contamination for long periods (Madnay et al., 2022; Wang et al., 2023). In detail, the levels of ASC in PGPB-treated plants in the present study, as the non-enzymatic component of the ASC-GSH cycle, were consistent with the activation of involved enzymes, especially APX, which catalyzes the reduction of H_2_O_2_ into H_2_O by ASC (Madany et al., 2020; AbdElgawad et al., 2023). Likewise, the activation of the ASC-GSH cycle in PGPB-treated plants was considered the main strategy for detoxifying ROS and preventing more cell damage in galaxolide-contaminated plants (Paradiso et al., 2008; Madnay et al., 2022). The rising levels of some antioxidant components (e.g. POX and GSH) in the present study in plants treated with PGPB and Se in polluted soils can be related to their known role in detoxification, transformation, and conjugation of environmental pollutants like galaxolide, apart from their inherent function in ROS scavenging (Madnay et al., 2022).

Despite all mentioned above, the lack of significant improvement of some components of antioxidant systems (e.g. ASC, DHAR, GR, and SOD) in response to Se nanoparticles in contaminated soils may be due to the sensitivity of these systems, especially the ASC-GSH cycle, which despite their defensive role in protecting cells from oxidative damage, they are susceptible to severe stress (El-Badri et al., 2022; Albqmi et al., 2023b). Therefore, it can be stated here that the antioxidant defense systems of Se-treated plants in the contaminated soils were not very active, at least not as much as PGPB-treated plants.

Our findings also indicated a piece of evidence for boosting the levels of polyphenols, flavonoids, total tocopherols, and total antioxidant capacity, in plants grown in contaminated soils, especially those treated with PGPB. These results are consistent with those of other studies that reported higher levels of antioxidant molecules in PGPB- and Se-treated crops under different environmental stress, in which they can adapt the plant to the stress conditions (Sonbarse et al., 2020; Yaghoubi Khanghahi et al., 2022b; Albqmi et al., 2023b), in particular through the protection of the photosynthetic system (AbdElgawad et al., 2014). Accordingly, it appears that high concentrations of these molecules in the present study, especially in PGPB-treated plants, were one of the leading protection mechanisms under galaxolide contamination.

Another active mechanism in plants under oxidative stress is the accumulation of proline (Meena et al., 2019) since this amino acid is known for its involvement in cell formation and its function in scavenging the free radicals and stabilizing the macromolecules (Matysik et al., 2002). The significant increase in proline accumulation in the combined treatment (PGPB+Se) in plants grown in contaminated soil can indicate the employing of this strategy by soybean plants in boosting the tolerance against stress caused by galaxolide, an improvement that was not significant in the individual application of the treatments compared to unfertilized plants. The increase in the synthesis of pyrroline-5-carboxylate synthase (P5CS), as one of the main enzymes involved in the synthesis of proline from the glutamate pathway, in the combined treatment in contaminated plants can be a confirmation of the improvement of proline level in this condition. Nevertheless, these findings cannot be extrapolated to all stress conditions since it has been proposed that the proline accumulation should not be utilized as a sole indicator of stress sensitiveness or tolerance but instead complemented with additional biochemical and physiological endpoints (Spormann et al., 2023). It is important to bear in mind the higher proline content in stressed plants could appear as a sign of high sensitivity to stress, acting as a warning that signals the imposition of stress without necessarily being involved in cell protection (Cia et al., 2012).

Moreover, the concentration of galaxolide in the plant leaves was decreased in plants treated with PGPB, Se, and PGPB+Se treatments in galaxolide-contaminated soil compared to the unfertilized plants. Increments in the contents of phytochelatins and metallothioneins (MTC) in the plants treated with combined treatment under galaxolide contamination, can confirm prior research, which recorded the activation of a complex network of detoxification strategies in plants under environmental pollution, via chelating of metal ions with phytochelatins and MTC in the cytosol, and thereafter, sequestrating into the vacuole (Zhao and Chengcai, 2011; Hasan et al., 2017). These defense molecules are structurally and functionally correlated to GSH and are proposed to be the product of the same biosynthetic pathway (Rodrigo et al., 2016), in which GSH can serve as the substrate for the biosynthesis of these molecules (Rauser, 1999). Therefore, the higher concentration of GSH in treated plants grown in polluted soils might be a reason for improving the levels of phytochelatins and MTC in plants. Likewise, the function of phytochelatins in improving the efficiency of GSH detoxification pathway in galaxolide-contaminated plants has been previously proved (Madnay et al., 2022). In addition, more increased activeness of glutathione S-transferases (GST) enzyme in PGPB-containing treatments in contaminated soil is consistent with other studies, in which GST was suggested as a defensive mechanism against oxidative damage by quenching the free radicals with the activeness of GSH in pollution-induced stress (Kumar and Trivedi, 2018; Madnay et al., 2022). These results indicated the regulatory function of such detoxifying metabolites in plants treated with PGPB+Se to regulate the ROS homeostasis in plants under galaxolide pollution.

## 5 Conclusions

The present research was carried out to address the active strategies of soybean plants under galaxolide contamination in soil when they were exposed to inoculation with plant growth-promoting Actinobacteria and supplementation with Se nanoparticles. Enhancement in the oxidative biomarker concentration and adverse consequences in some physiological and biochemical traits in plants revealed oxidative damage for those grown in galaxolide-induced stress. Nevertheless, the prominent potential of beneficial Actinobacteria and Se nanoparticles was illustrated in higher production of plant biomass, advancement in photosynthetic pigments and capacity, activation of the photorespiratory pathway, biosynthesis of metabolites, and activation of antioxidant pathways in galaxolide-stressed plants. Hence, it could conceivably answer the presented hypothesis in this study that the soybean plants have profited from both PGPB and Se treatments, however, their synergetic impacts (PGPB+Se treatment) were more apparent under oxidative stress induced by galaxolide contamination.

## Data availability statement

The original contributions presented in the study are included in the article/supplementary material. Further inquiries can be directed to the corresponding author.

## Author contributions

RH and FA conceived the study. FA and RH conducted the experiments. FA and RH analyzed the results and performed the statistics and illustrations. FA and RH wrote the manuscript draft. All authors contributed to the article and approved the submitted version.
